# Neurorestorative properties of ibogaine: linking multi-receptor affinities to remyelination and metabolic restoration

**DOI:** 10.1017/neu.2026.10059

**Published:** 2026-02-13

**Authors:** Tanya Calvey, Demi Govender, Gavin R. Owen, Nancy Tumba, Dirk Lang, Bernard Lerer, Dan J. Stein, Steven Shoptaw

**Affiliations:** 1 Department of Human Biology, University of Cape Town, South Africa; 2 Neuroscience Institute, University of Cape Town, South Africa; 3 HIV Pathogenesis Research Unit, Faculty of Health Sciences, University of the Witwatersrand, Johannesburg, South Africa; 4 Infectious Diseases and Oncology Research Institute (IDORI), Faculty of Health Sciences, University of the Witwatersrand, Johannesburg, South Africa; 5 Centre for Psychedelic Research, Hadassah Medical Center, Hebrew University, Jerusalem, Israel; 6 SAMRC Unit on Risk & Resilience in Mental Disorders, Department of Psychiatry, University of Cape Town, South Africa; 7 Department of Family Medicine, University of California Los Angeles, USA

**Keywords:** ibogaine, myelin sheath, opioid-related disorders, multiple sclerosis, traumatic brain injury

## Abstract

Ibogaine is a psychedelic alkaloid without an approved indication. Observational clinical research shows linkages between single administration of ibogaine and relief of symptoms of neuropsychiatric conditions including substance use disorder, multiple sclerosis (MS), and traumatic brain injury. Ibogaine has multi-receptor actions, but the neurobiological mechanisms underlying such putative effects are unknown. Here we review and discuss the relevant literature, focusing on remyelination and metabolic restoration. We provide evidence that ibogaine upregulates markers of myelination following opioid administration; that conditions such as opioid use disorder, MS and traumatic brain injury are characterised by white matter pathology; that decreased myelination is related to dysregulated metabolic homeostasis, ischaemia and hypoxia which may also play a role in these disorders. We conclude that multi-receptor actions of ibogaine, especially its affinities for the NMDA, kappa opioid and sigma receptors, in turn account for reduction in excitotoxicity, metabolic regulation, lasting neuroplasticity and immunomodulation that facilitates neuronal repair and remyelination providing a rationale for future investigation of its use as a therapeutic agent for these common central nervous system disorders.

## Graphical abstract

**Figure:**
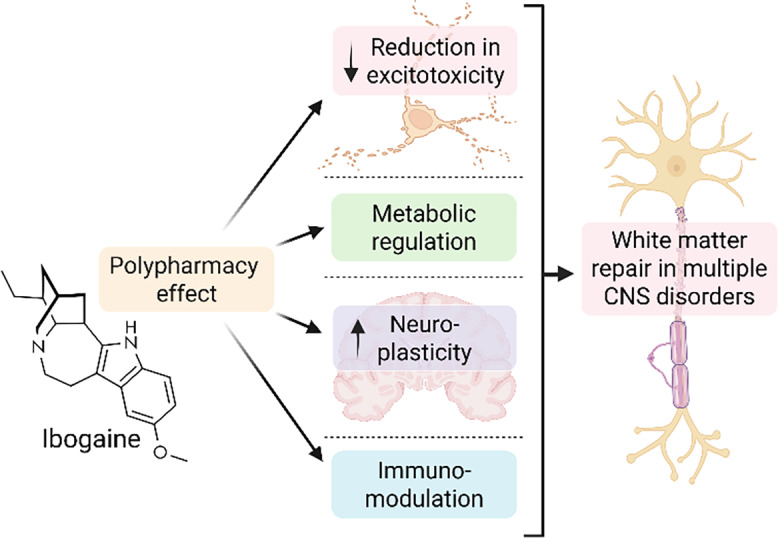

Summations

- Preclinical research and a case study of multiple sclerosis have indicated that ibogaine may increase myelination and reduce white matter lesion volume. These activities would support ibogaine as a compound of interest to facilitate neuronal repair in diseases of the central nervous system where white matter pathology is a central feature, e.g. opioid use disorders, traumatic brain injury and multiple sclerosis.
- Open-label observational data indicate that ibogaine may improve symptoms of opioid use disorder and traumatic brain injury further highlighting the importance of understanding the neuropharmacology of ibogaine as a possible treatment for central nervous system injury.
- Ibogaine has affinity for several neurotransmitter systems including the opioidergic, glutamatergic, serotonergic and sigma receptor systems. Studies of single dose or repeated doses of ibogaine may demonstrate ways the drug can support remyelination, restore metabolic homeostasis and decrease neural excitotoxicity.

Perspectives

- Human observational and animal data provide sufficient rationale for future controlled trials of therapeutics for opioid use disorders, multiple sclerosis and traumatic brain injury.
- Future controlled trials of ibogaine and its analogues could test the mechanism of the drug for white matter repair using diffusion-weighted imaging.
- Ibogaine administration requires careful patient screening and monitoring to address risks for cardiac arrhythmia; co-treatment with magnesium may mitigate some of this risk.

## Introduction

Ibogaine is the primary alkaloid in *Tabernanthe iboga* root bark cultivated in central Africa. It has been used in traditional African medicine for centuries for its psychedelic properties and more recently for its well-established anti-addictive properties. Open label and observational studies link Ibogaine administration with reductions in drug cravings, in symptoms of withdrawal, and in return to opioid use (Barsuglia *et al*., 2018; Calvey and Howells, 2018; Mash *et al*., 2000; 2018).

Ibogaine has a multi-receptor profile and the metabolite, noribogaine, remains available in blood plasma for 7 days (Glue *et al*., 2015a) (for ranked receptor affinities, see Table 1). Both ibogaine and noribogaine are kappa opioid receptor agonists (noribogaine > ibogaine) and weak mu opioid receptor antagonists (Maillet *et al*., 2015; Ona *et al*., 2023; Mash, 2023b). Ibogaine has a modulating effect on the dopaminergic system via its weak affinity for the dopamine transporter (Wells *et al*., 1999; Baumann *et al*., 2001) and its potential ability to repair dopamine transporter folding (Bhat *et al*., 2020). Ibogaine and noribogaine have weak affinities for several serotonin receptors including the serotonin 2A and 1A receptors, are potent non-competitive inhibitors of the serotonin transporter (noribogaine > ibogaine) and both inhibit the transport function of the vesicular monoamine transporter 2, which may contribute to its antidepressant and antiaddictive properties (Ray, 2010; Bulling *et al*., 2012; Ona *et al*., 2023; Hwu *et al*., 2025). Ibogaine is also a potent noncompetitive inhibitor of nicotinic alpha3beta4 receptors (ibogaine > noribogaine), a competitive *N*-methyl-D aspartate (NMDA) receptor antagonist (ion channel blocker; ibogaine > noribogaine), has a moderate to high affinity for sigma 2 (ibogaine > noribogaine) and a moderate to low affinity for sigma 1 (Popik *et al*., 1994; Bowen *et al*., 1995; Mash *et al*., 1995; Arias *et al*., 2010; Ona *et al*., 2023; Mash, 2023a).

**Table 1.** Ranked binding site affinities of ibogaine and noribogaine neuronal repair and remyelination (Ki; IC50; µM)

Target and ranking	Affinity ibogaine	Affinity noribogaine	Action	Repair mechanism
1. Kappa opioid receptor	2.1–13.8	0.61–0.96	Biased agonist (inhibits beta -arrestin) (Maillet *et al*., 2015; Glick *et al*., 1999)	Remyelination (Du *et al*., 2016; Mei *et al*., 2016)
2. NMDA receptor	1–5.6	15–31.4	Channel blocker (Chen *et al*.,^1996^; Mash *et al*., 1995; Popik *et al*., 1994)	Reduced excitotoxicity (Wang *et al*., 2022)
3. Sigma-2 receptor	0.2–0.3	Inactive	Agonist (Bowen *et al*., 1995; Mach *et al*., 1995)	Neuronal repair (Terada *et al*., 2018; Vázquez-Rosa *et al*., 2018; Yi *et al*., 2017)
4. SERT	0.2–2.5	0.04–0.9	Serotonin reuptakeblocker (Staley *et al*., 1996; Bulling *et al*., 2012; Coleman *et al*., 2019)	May facilitate the immunomodulatory function of serotonin.Improve mood and pain (Bulling *et al*., 2012)
5. 5HT2A receptor	14	32.5–92.5	Weak agonist (Helsley *et al*., 1998; Ray, 2010)	Immunomodulatory (Yu *et al*., 2008) Structural neural plasticity (Ly *et al*., 2018)
6. Sigma 1	5.8	Inactive	Agonist (Ray, 2010)	Promotes oligodendrogenesis (Song *et al*., 2023)
7. Mu opioid receptor	2–3	0.68–13	Partial, mixed agonist, antagonist (Glick *et al*., 1999; Antonio *et al*., 2013)	Unknown but may influence kappa opioid receptor function (Govender *et al*., 2024)
8. α3β4 nAChR	0.22	6.2	Antagonist (Arias *et al*., 2010)	Reduced neurotransmission (Kanasuwan *et al*., 2023)

*Note*: nAChR, nicotinic acetylcholine receptors; SERT, serotonin transporter, 5HT2A = serotonin 2A receptor.

Interest in ibogaine’s treatment potential for disorders other than opioid use disorder include several case studies and uncontrolled trials that link ibogaine administration with reductions in symptoms of post-traumatic stress disorder (PTSD) (Barsuglia *et al*., 2018; Cherian *et al*., 2024a), multiple sclerosis (MS)(Chen *et al*., 2025), neuropathic pain disorder (Dickinson *et al*., 2023), traumatic brain injury (Cherian *et al*., 2024a) and Parkinson’s disease (Erny *et al*., 2026). As this single compound, administered only once, is observed to reduce symptoms of several neuropsychiatric conditions, its mechanism of action is an important question, which appears to be linked to ibogaine’s multi-receptor activity that contributes to neural plasticity (Ly *et al*., 2018). Recent research has indicated ibogaine’s ability to upregulate protein and gene expression in markers of white matter remyelination (CNP 2’, 3’-cyclic nucleotide 3’-phosphodiesterase; Myelin Basic Protein) in the internal capsule following chronic morphine administration (Govender *et al*., 2024) and reduce white matter lesion volume in a case study of MS (Chen *et al*., 2025). Ibogaine’s potential to treat white matter pathology and facilitate its repair is an important potential mechanism of action that we explore in detail.

Several neurological and psychiatric disorders are associated with demyelination of neurons in the central nervous system including MS, progressive multifocal leukoencephalopathy, schizophrenia and substance use disorders, particularly opioid use disorders. Demyelination in CNS white matter occurs when there is a loss of myelin and a failure of remyelination (Miller and Mi, 2007). There are both primary and secondary demyelinating disorders that are categorised according to whether degeneration occurred before or after the myelin loss or axonal injury (Love, 2006). Primary demyelinating disorders include MS where there is myelin loss prior to the axonal damage. The secondary demyelinating diseases include diseases that cause myelin loss or white matter changes due to viruses, vitamin deficiencies, genetic disorders, radiation or toxic drug exposure such as opioid or cocaine use (Ryan *et al*., 2014; Jangir *et al*., 2023).

Very little is known about ibogaine’s effects on myelination or the processes that facilitate remyelination. This article will focus on the molecular properties of ibogaine that may facilitate remyelination. Processes that facilitate axonal repair may improve clinical outcomes in MS, substance use disorder and traumatic brain injury. To this end, we review and discuss the molecular biology of white matter, white matter pathology associated with MS, opioid use disorder and traumatic brain injury as well as the potential mechanisms of action of ibogaine related to myelination and the processes that may facilitate remyelination.

## Composition of white matter and myelination

White matter contains myelinated axons and the associated microglia, oligodendrocytes, and astrocytes. The myelin around the axons of nerve fibres allows for faster and more efficient propagation of electrical signals, referred to as saltatory nerve conduction. Another function of the myelin sheath, which comprises both oligodendrocytes and myelin, is to provide metabolic support for the axon which is essential for survival (Linder *et al*., 2008).

Myelin is composed of lipids and proteins which provide structural support and can be used as markers of myelination (Linder *et al*., 2008; Oberoi *et al*., 2019). Myelin basic protein (MBP) is produced by the oligodendrocyte prior to myelin covering the axon as it is important for membrane adhesion and the cytoskeletal structure of myelin (Boggs, 2006; Harauz and Boggs, 2013). During remyelination MBP may facilitate secretion of neurotropic growth factor and brain derived neurotrophic factor (BDNF) (Harauz and Boggs, 2013) and increase oligodendrocyte proliferation. CNP 2’, 3’-cyclic nucleotide 3’-phosphodiesterase (CNPase) makes up 4% of the proteins present in myelin and is present in the cytoplasm of oligodendrocytes (Verrier *et al.*, 2013). During the early stages of oligodendrocyte differentiation CNPase is essential and has been associated with compacting the myelin layers on axons (Maier *et al*., 2008). Proteolipid protein (PLP) is the most abundant protein in CNS myelin (Laule *et al*., 2007). It is hydrophobic, spans the myelin membrane and adheres the myelin lamellae creating compact concentric layers for myelin stability. There are two additional myelin associated proteins that are present in the CNS but in very small quantities. These are myelin associated glycoprotein (MAG) and myelin oligodendrocyte protein (MOG) (Wang *et al*., 2018) and Nogo-A (Chen *et al*., 2000). MAG and Nogo-A (also known as Reticulon 4) have been shown to act as regulators of neuronal plasticity and as inhibitors of axonal regeneration after traumatic brain injury (McKerracher and Rosen, 2015).

Myelination is the process of creating myelin around an axon whereby oligodendrocytes spiral-wrap their plasma membranes around axons (Figlia *et al*., 2018) and upregulate the expression of myelin associated proteins such as MBP or CNPase (Hattori *et al*., 2014; Bothwell, 2017). This process occurs during embryonic development and during learning (Monje, 2018; Stadelmann *et al*., 2019). Myelination also takes place following a demyelinating event such as a physical injury or by exposure to toxic substances and facilitates axon repair (Franklin and Ffrench-Constant, 2008; Herford, 2017) and is termed remyelination.

Remyelination is vital for axonal survival and is crucial for central nervous system (CNS) development and recovery following insult. There are two mechanisms of new myelin formation following demyelination – one involving the generation of new oligodendrocytes, and one involving existing oligodendrocytes (Franklin *et al*., 2021). The former process occurs in two steps. First, adult oligodendrocyte progenitor cells (OPCs) are activated and recruited to sites of demyelination. The second step is the differentiation of OPCs into remyelinating oligodendrocytes. The oligodendrocytes will then contact the demyelinated axon and synthesise myelin which forms concentric layers around the axon (Figlia *et al*., 2018). This membrane will be thinner than the original myelin sheath as this is a distinguishing feature of remyelination especially on larger axons (Blakemore, 1974; Franklin and Ffrench-Constant, 2008; Franklin and Simons, 2022). The alternative mechanism of myelination is when mature surviving oligodendrocytes contribute to remyelination in humans (Falcao *et al*., 2018; Jakel *et al*., 2019).

As myelin is lipid-based, an important metabolic component of de- and remyelination is lipid recycling, that is, lipid catabolism, synthesis and export. Myelin debris and inflammation inhibit oligodendrocyte differentiation (Kotter *et al*., 2006). During repair of demyelinated lesions, there is lysosomal degradation of myelin lipids and proteins by phagocytes. Remyelination requires recycling of lipids for export to oligodendrocytes (Berghoff *et al*., 2022).

## Diseases of white matter potentially treatable with ibogaine

### Opioid use disorder

Substance use, particularly opioid use, is associated with reductions in cortical volume as well as altered white matter structure and integrity (Wollman *et al*., 2015; Moningka *et al*., 2019; Ndlovu *et al*., 2021). Chronic opioid administration has been found to decrease MBP expression in rats (Fan *et al*., 2018; Quintanilla *et al*., 2023). In humans, the extent of white matter damage worsens with an increase in the duration of exposure to opioids including methadone replacement therapies (Wang *et al*., 2011; Bora *et al*., 2012; Li *et al*., 2013).

Opioid-associated leukoencephalopathy results from axonal injury with secondary demyelination (Alturkustani *et al*., 2017). Heroin spongiform leukoencephalopathy is characterised by spongiform degeneration of the white matter with large vacuoles, axonal spheroids and areas that have a loss of myelin, astrocytes, oligodendrocytes and axonal density (Alturkustani *et al*., 2017; Büttner *et al*., 2000; Jee *et al*., 2009; Kass-Hout *et al*., 2011; Jangir *et al*., 2023). Leukoencephalopathy associated with opioid and cocaine use induces histological changes in human white matter that is characteristic of hypoxic-ischaemic pathophysiology. Alturkustani *et al*. (2017) report on these histological changes and describe how both heroin and cocaine induce cerebral hypoperfusion, heroin through reduced respiration and cardiac output and cocaine through vasoconstriction.

Four open label trials of *n* = 102 (Mash *et al*., 2018), 30 (Brown and Alper, 2018), 14 (Noller *et al*., 2018) and 73 (Davis *et al*., 2018) provide observational data that demonstrate single dose administration of ibogaine corresponds with reductions of opioid use, specifically prescription opioids and heroin. A single administration of ibogaine is able to improve opioid craving, insomnia and mood as well as reduce drug consumption (Mash *et al*., 2018; Noller *et al*., 2018; Mash, 2023a) for several weeks after treatment. Ibogaine also modulates the analgesic effects and tolerance to morphine and reduces symptoms of opioid withdrawal (Cherian *et al*., 2024b).

Ibogaine administration for opioid use disorder requires careful patient screening and monitoring due to safety issues (Cherian *et al*., 2024b; Kock *et al*., 2022). Ibogaine is a potent inhibitor of the human Ether-à-go-go-Related Gene (hERG) potassium channels which can lead to QT prolongation and arrhythmias. In the mid-1990s, there was one fatality in an FDA-approved clinical trial yet several controlled phase 1 and 2 trials have been conducted since without any serious adverse events (Glue *et al*., 2015a, 2015b, 2016; Prior and Prior, 2014; Cherian *et al*., 2024b). When ibogaine is administered by trained professionals that follow health and safety protocols, the likelihood of fatality seems to be low (Cherian *et al*., 2024b). This being said, 33 deaths following ibogaine administration were reported in the literature between 1990 and 2021 (Mash, 2023a, b) and since then, additional deaths at clinics in Mexico and Canada have been reported in the media (Busby, 2024). The incidences in the literature were in environments where either the facilitators were medically inexperienced or the individuals were unsupervised so may have consumed additional substances (Mash, 2023a). Contributing factors include CYP2D6 drug interactions, polydrug use, alcohol withdrawal, concurrent methadone and benzodiazepine use, undiagnosed CVS disease and potassium and magnesium abnormalities (Cherian *et al*., 2024b; Kock *et al*., 2022). Opioid withdrawal itself can lead to electrolyte abnormalities (Luz and Mash, 2021; Mash, 2023a). Cherian *et al*. (2024a) administered ibogaine together with magnesium (1–2 h before and 12 h after ibogaine administration) to 30 patients and no adverse cardiac events took place. This being said, exclusion for cardiac risk in ibogaine treatment may limit many of those with OUD, traumatic brain injury or MS. Analogues with reduced cardiotoxicity are being developed to this end (Havel *et al*., 2024).

### Multiple sclerosis

MS is the most common demyelinating disorder of the CNS and is characterised by focal areas of inflammation, axonal damage, blood–brain barrier breakdown and progressive neurological deficits (Papiri *et al*., 2023; Chen *et al*., 2025). MS lesions are regions of inflammatory myelin breakdown and reactive gliosis. MS follows a relapsing-remitting (RRMS) or progressive course, which is further distinguished between primarily (PPMS) or secondarily progressive (SPMS). Proportions of active versus inactive lesions differ between RRMS and P/SPMS. Active lesions have a significant inflammatory component and increased permeability of the BBB in comparison to inactive lesions and active lesions are more common in RRMS (Love, 2006).

Remyelination takes place in RRMS but the process is terminated early leaving thinly myelinated axons termed ‘shadow plaques’. OPCs are not recruited and thus remyelination is only facilitated by mature oligodendrocytes along with the myelin debris from demyelination further inhibiting the remyelination progress (Patrikios *et al*., 2006; Patani *et al*., 2007; Frischer *et al*., 2015; Zhao and Jacob, 2023). Therefore, recovery likely targets and activates mature cells. Remyelination potential is lower in more progressive forms of MS (Franklin and Gallo, 2014).

Treatments targeting mitochondrial dysfunction may assist with recovery of remyelination in MS. Mitochondrial dysfunction is a key feature of early MS and is caused by a reduction in cerebral blood flow (Franklin and Gallo, 2014; Mozafari *et al*., 2025). The resulting ischaemia activates hypoxia cascades that alters the number, functioning and morphology of mitochondria. Treatments targeting ischaemia preconditioning such as ‘remote ischaemic preconditioning’ may improve gait in patients with MS (Chotiyarnwong *et al*., 2020). Treatments targeting mechanistic target of rapamycin (mTOR) are able to increase myelination in animal models of MS (Chen *et al*., 2000; Sanadgol *et al*., 2020).

Ibogaine may be able to reduce white matter lesion volume and improve functioning in MS. Chen *et al*. (2025) present two case studies with patients receiving ibogaine HCl treatment for MS. Patient A had RRMS and patient B had SPMS. Following ibogaine, magnetic resonance imaging revealed Patient A had a 72% reduction in white matter lesion volume and a mean decrease in Apparent Diffusion Coefficient, which suggests remyelination, improved neural integrity and reduced inflammation. Symptoms of MS, related to fatigue, motor and bladder function resolved and a year after treatment patient A ran an ultra-marathon. In patient B with SPMS, imaging revealed a mean increase in Apparent Diffusion Coefficient although levels of pain and physical function improved albeit to a lesser extent in comparison to patient A’s recovery.

### Traumatic brain injury

Traumatic brain injury (TBI) is injury to the brain from an external traumatic event that leads to loss of neural function and tissue homeostasis due to impairment of the blood–brain barrier, osmotic imbalance, inflammatory processes, oxidative stress, excitotoxicity, and apoptotic cell death (Freire *et al*., 2023). This leads to reduced cerebral blood flow as well as a change in brain structure and function and cognitive changes include reductions in attention, processing speed and executive functions (Cherian *et al*., 2024a). Following the external injury, secondary injury takes place via various mechanisms, including ischaemia, excitotoxicity, oxidative stress, inflammation, oedema and mitochondrial dysfunction and can be influenced by factors such as systemic hypotension, hypoxaemia, increased intracranial pressure, and metabolic imbalances (Sanadgol *et al*., 2020).

Animal models of TBI have uncovered molecular and structural mechanisms related to white matter pathology including myelin reduction (Ozen *et al*., ), axonal damage and injury (Kilbourne *et al*., 2009; Zhang *et al*., 2018), as well as white matter gliosis (Bashir *et al*., 2020). TBI can also affect neurotransmitter metabolism which has been implicated in the pathogenesis of neuropsychiatric disorders following injury (Franklin and Gallo, 2014). BDNF is released following TBI and in rats, levels are increased in cerebral fluid between 1 and 6 h after a brain trauma (Wang *et al*., 2014). The mTOR pathway is also a possible target for treatment following TBI (Movahedpour *et al*., 2022).

In an open-label study, Cherian *et al*. (2024a) administered ibogaine HCl and magnesium to 30 patients with mild TBI following military service. Treatment resulted in significant immediate and sustained improvements in functioning measured with the World Health Organization Disability Assessment Schedule with the greatest effect sizes noted for pre- to post-cognition domains (Cohen’s *d* = 0.96), particularly in processing speed and executive function. There were also significant improvements in measures of symptoms of PTSD, depression and anxiety. The authors comment that theirs is the first study to report on a single administration of a drug improving chronic disability related to repeated TBI.

## Possible mechanisms of action of ibogaine

**Figure 1.**
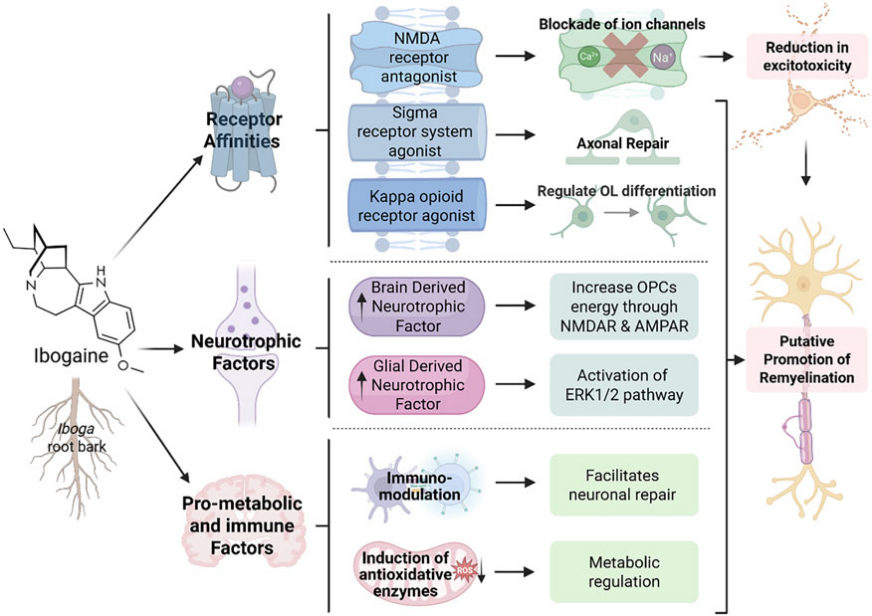
The potential neurorestorative effects of ibogaine on processes of remyelination. This figure depicts the metabolic factors (Bentura *et al*., 2024; Paškulin *et al*., 2006; 2012) neurotrophic factors (He *et al*., 2005; Marton *et al*., 2019) and receptors influenced by ibogaine and its metabolite, noribogaine (Ona *et al*., 2023; Mash, 2023a). These may have subsequent effects on either oligodendrocytes or OPCs (Small *et al*., 1998; Xiao *et al*., 2011; Ma *et al*., 2024), which are the myelin producing cells of the central nervous system, or repair pathways such as ERK1/2 or potentially mTOR (Figlia *et al*., 2018; Ly *et al*., 2018; Paškulin *et al*., 2006; Olson, 2022).

White matter repair is metabolically intensive. Myelin is lipid-based; repair of myelin requires neural plasticity, reduced inflammation and a pro-metabolic environment. There are several molecular mechanisms of action that may contribute to ibogaine’s potential as a treatment agent in diseases that may respond to neuronal repair and remyelination such as OUD, MS, and TBI (see Figure 1). Ibogaine has multi-receptor binding affinity, and is likely to regulate multiple neurotransmitter systems, improve metabolic homeostasis, and increase cerebral blood perfusion. Ibogaine is also able to upregulate processes of remyelination (Chen *et al*., 2025; Govender *et al*., 2024) and possible molecular mechanisms are detailed below.

### Receptor affinities specific to the process of myelination

Ibogaine is a kappa opioid receptor agonist and noribogaine is a potent agonist (ibogaine: 2.1–13.8 µM; noribogaine: 0.61–0.96 µM) (Glick *et al*., 1997; Maillet *et al*., 2015; Mash, 2023a; Mash, 2023b; Havel *et al*., 2024) (for ranked receptor affinities, see Table 1). The kappa opioid receptor has been shown to influence remyelination through oligodendrocytes and their precursor cells. The kappa opioid receptor is expressed on OPCs and is an important regulator of oligodendrocyte differentiation (Du *et al*., 2016; Mei *et al*., 2016; Dalefield *et al*., 2022).

The findings of Govender *et al.* (2024) suggest that combined morphine and ibogaine administration augments the expression of markers of myelination (MBP and CNPase) more than either substance on its own. This may point to an unexplored relationship between the mu and kappa opioid receptors and their roles in myelination. Maillet *et al*. (2015) found that noribogaine functionally inhibits the recruitment of beta arrestin by dynorphin. This mechanism not only explains noribogaine’s ability to reduce opioid tolerance but suggests that the action of the kappa opioid receptor is further enhanced. Understanding the interaction between noribogaine’s direct effects on dynorphin and myelin could lead to new therapeutic approaches for neurological disorders, including MS.

Ibogaine is a sigma-2 receptor agonist and possesses a selective, moderate-to-high affinity for sigma 2 sites (ibogaine >> noribogaine) (Bowen *et al*., 1995; Mach *et al*., 1995). The sigma 2 receptor is involved directly and indirectly in neuronal repair and compounds targeting the sigma 2 receptor are therapeutic agents currently in clinical trials for treating Alzheimer’s disease and schizophrenia (Vázquez-Rosa *et al*., 2018). A sigma 2 modulator decreased axonal degeneration, enhanced survival of cortical neurons and oligodendrocytes and preserved cognitive function after TBI in mice (Vázquez-Rosa *et al*., 2018). The mechanism of this repair is thought to be related to the sigma 2 receptor’s ability to regulate cholesterol transporter-regulating protein Niemann-Pick1 availability in cells, which in turn alters intracellular cholesterol use for cellular membrane plasticity. Other studies have found that the sigma 2 receptor plays an important role in neurite outgrowth (Terada *et al*., 2018) and has neuroprotective and anti-inflammatory properties (Yi *et al*., 2017). Ibogaine has a moderate to low affinity for sigma 1 (Ray, 2010) a receptor with known neuroprotective and pro-metabolic properties (Ryskamp *et al*., 2019). It is also involved in lipid transport and has been found to promote oligodendrogenesis and white-matter functional recovery in mice (Song *et al*., 2023). Future studies should focus on the sigma receptor system’s role in remyelination and axonal repair.

Chen *et al*. (2025) indicate that ibogaine has a greater treatment effect on RRMS than SPMS suggesting that it targets mechanisms involved in active lesions so improving energy metabolism, reducing inflammation and facilitating myelination. The fact that there is less of an effect in SPMS suggests that these mechanisms have halted in inactive lesions. It also suggests that ibogaine is able to activate mature oligodendrocytes. The initial sigma 2 affinity of ibogaine could activate this process followed by the long-acting effects of noribogaine which may promote the development of new oligodendrocytes due to its kappa opioid receptor affinity. The findings of Chen *et al*. (2025) might also suggest an effect of ibogaine on the restoration of neuronal connections after disruption, possibly involving regulation of neurite growth inhibitory protein expression (MAG, Nogo-A) or their receptors. Further research into these possible molecular mechanisms would be of great value to the field.

Ibogaine is also an NMDA receptor antagonist (Popik *et al*., 1994; Mash, 2023a). The blockade of NMDA ion channels would improve the effects of excitotoxicity in MS and TBI (see Figure 1). Excitotoxicity is a driver of neurodegeneration in MS and ketamine, a psychedelic NMDA antagonist is able to reduce inflammation caused by neurotoxicity (Anchesi *et al*., 2025). Mandal *et al*. (2024) demonstrated that ketamine is able to protect hippocampal neurogenesis during inflammation induced by pro-inflammatory cytokines such as IL-1*β*. Ketamine has also been found to reduce demyelination and facilitate remyelination in a cuprizone model of MS in rats (Wang *et al*., 2022). Excitotoxicity is also a driver of neurodegeneration following TBI. Imbalanced concentrations of extracellular glutamate following TBI induce the activation of Na+ and Ca2+ channels in the cell membrane, resulting in increased release of glutamate by the cell, overstimulating NMDA receptors, leading to more Ca2+ influx, thus establishing a detrimental looping that ultimately results in excitotoxic cell death (Luo *et al*., 1993; Atlante *et al*., 1997).

Ibogaine is a weak serotonin 2A receptor agonist (ibogaine > noribogaine) and does not produce head twitch/shake response in rodents unlike classic psychedelics yet some of its effects are due to interaction with serotonin 2A receptors (Helsley *et al*., 1998; Villalba *et al*., 2024). A single dose of noribogaine promotes structural plasticity by increasing dendritic branching complexity in rats and this effect is blocked by ketanserin, a serotonin 2A antagonist (Ly *et al*., 2018). This ability to promote structural neural plasticity is one likely mechanism of lasting changes from a single dose.

Serotonin 2A receptor agonists can also produce potent anti-inflammatory effects by modulating immune-related signalling pathways and do so at doses lower than those required for psychedelic effect (Yu *et al*., 2008; Anchesi *et al*., 2025). These anti-inflammatory actions may be able to remove inhibitory signals on oligodendrocyte maturation, thus facilitating the natural repair process (Anchesi *et al*., 2025). Research into ibogaine’s effects on immune function is lacking but early studies suggest an immunomodulatory effect. House *et al*. (1995) found in vitro exposure to ibogaine resulted in a dose-related suppression of T-cell, B-cell, and natural killer cell functions and ibogaine has also been found to reduces colonisation by *Candida albicans* infection in mice (Yordanov *et al*., 2005).

The neurological and psychiatric treatment potential of ibogaine may lie in all its receptor affinities now termed the ‘polypharmacy effect’ (Ona *et al*., 2023) or ‘matrix pharmacology’ (Hwu *et al*., 2025). For example, TBI can also affect neurotransmitter metabolism by affecting the synthesis, release, reuptake, and metabolism of neurotransmitters such as serotonin, dopamine, GABA and glutamate. These alterations can impact mood, behaviour and cognitive function, and may contribute to the development of neuropsychiatric disorders following the injury (Freire *et al*., 2023). Given ibogaine’s affinities for so many of the neurotransmitter systems, improvements in neurotransmission may provide a putative mechanism of action for improving cognitive function as in TBI (Cherian *et al*., 2024a) and MS (Chen *et al*., 2025) and as in mood in PTSD (Barsuglia *et al*., 2018; Cherian *et al*., 2024a) and OUD (Mash *et al*., 2018).

### Neurotrophic factors

BDNF, from the neurotrophin family of growth factors is essential for brain development, learning, memory and neural plasticity (Brigadski and Lebmann, 2020; Xiao, 2023). It regulates neural plasticity through myelination, neuronal morphology and architecture and synaptic changes. BDNF and myelination are strongly associated in development and after injury (Fletcher *et al*., 2018). BDNF knockout mice showed decreased expressions of MBP and decreases in mRNA expression of MBP and PLP although these mice died prematurely (Cellerino *et al*., 1997; Djalali *et al*., 2005). The evidence suggests that BDNF is a promyelinating factor (Fletcher *et al*., 2018) but there are other mechanisms that BDNF affects to enhance remyelination.

Increased BDNF activates NMDA and AMPA receptors on OPCs which increases the OPCs energy supply (Small *et al*., 1998; Carvalho *et al*., 2008; Xiao *et al*., 2011; Ma *et al*., 2024). This increase in energy allows for survival, proliferation and differentiation of the OPCs. Direct infusions or cell therapy of BDNF in stroke and spinal cord injury increased BDNF and subsequently MBP, PLP, CNP and MOG proteins (Marton *et al*., 2019; McTigue, 1998; Ramos-Cejudo *et al*., 2015). Several studies have demonstrated ibogaine-induced increases in BDNF mRNA in multiple brain regions typically peaking at 24h post-administration with less robust effects on protein levels (Marton *et al*., 2019). This suggests that the underlying mechanism behind these remyelination effects seen in rats (Govender *et al*., 2024) and humans (Chen *et al*., 2025) could be due to the effect of ibogaine on BDNF.

Glial derived neurotrophic factor (GDNF) has similar remyelination effects as BDNF but acts through the ERK1/2 pathway. Ibogaine upregulates GDNF mRNA expression (He *et al*., 2005; Carnicella *et al*., 2010; Marton *et al*., 2019) and this has been shown to act on GFR*α*1 to stimulate ERK1/2 phosphorylation as demonstrated by He *et al*. (2005) in human neuroblastoma cells. Sustained activation of the ERK1/2 pathway in OPCs improves remyelination and increases the myelin thickness in adult oligodendrocytes (Fyffe-Maricich *et al*., 2013; Jeffries *et al*., 2016). The ERK1/2 phosphorylation is also essential for the maintenance of myelin (Ishii *et al*., 2014). Analogue, oxa-noribogaine induces long-term elevation of GDNF and BDNF in the prefrontal cortex, nucleus accumbens and ventral tegmental area of rats, an effect shown to be mediated by the kappa opioid receptor (Havel *et al*., 2021). This line of research is promising because it may clarify how the opioidergic system drives the remyelination potential of noribogaine and its safer analogues.

### Anti-ischaemic and pro-metabolic properties

Ibogaine administration leads to lasting effects on brain metabolism as shown in a recent Positron Emission Tomography (PET) study in rats (Bentura *et al*., 2024). Ibogaine also reduces oxidative damage and lowers basal metabolic needs by increasing efficacy of antioxidative systems (Paškulin *et al*., 2012). It also induces catabolic enzymes which reset metabolic equilibrium (Paškulin *et al*., 2006) and may protect against ischaemic pathogenesis in OUD, TBI, and MS as listed above. It has been observed in several disease models that the high energy consumption of nerve cells might render them particularly vulnerable to impaired energy production (Papiri *et al*., 2023). Myelination is also especially energy intensive as it is predominantly lipid-based and most lipids, including cholesterol, are synthesised de novo by myelinating cells in the CNS (Jurevics and Morell, 1995).

The mTOR pathway is implicated in the pathophysiology of OUD, MS, and TBI and is another possible therapeutic target for ibogaine. Neuronal mTOR function is influenced by the activity of growth factors and NMDA receptors and ketamine’s antidepressant properties are mediated by the mTOR signalling pathway (Abelaira *et al*., 2017). There is an increase in BDNF production following treatment with ketamine and classic psychedelics and BDNF binds to TrkB, resulting in mTOR activation (Olson *et al*., 2022). Both noribogaine and ketamine promote structural and functional neuroplasticity that is likely mediated by mTOR (Ly *et al*., 2018). mTOR facilitates anabolism, cell growth and lipid synthesis. It increases lipid synthesis by enhancing the function of Sterol Regulatory Element-Binding Protein (SREBP) transcription factors (Peterson *et al*., 2011; Jeon and Osborne, 2012). Ibogaine is lipophilic and accumulates in adipose tissue (Hough *et al*., 1996) which suggests an important role in lipid metabolism. Further research into ibogaine’s effects on the mTOR signalling pathway would be of great value to the field.

Ibogaine also potentially increases cerebral blood flow. Barsuglia *et al*. (2018) conducted the first human neuroimaging study of ibogaine administration, a case study on a 31-year-old male with treatment-resistant alcohol use disorder and PTSD. The patient was treated in a 4-day programme that included administration of ibogaine HCl (17.9 mg/kg) and 5-MeO-DMT. Single Photon Emission Computed Tomography (SPECT) scanning was performed on Days 1 and 4. There were significant increases in cerebral blood perfusion compared to baseline and lasting improvements in symptoms of alcohol use disorder and PTSD following treatment with ibogaine HCl and 5-MeO-DMT. This treatment effect cannot be attributed to ibogaine alone yet increase of blood flow would improve the metabolic environment and facilitate tissue repair and remyelination.

Ibogaine has several observational data reports that describe promising signal for relieving symptoms of opioid use disorders (Mash *et al*., 2018), MS (Chen *et al*., 2025) and mild TBI (Cohen’s d effect sizes 0.74–0.96) (Cherian *et al*., 2024a). Future controlled trials are urgently needed to confirm and extend these early findings and to assess ibogaine’s safety and efficacy in treating brain disease when compared to a standard of care. To the extent that ibogaine has value as a treatment for these conditions, its mechanism may link best to its upregulation of myelination processes and neuronal repair.

The temporal aspect of repair is important due to the differing receptor affinities of ibogaine and noribogaine (see Table 1). Ibogaine HCl administration results in immediate (24 h post administration) upregulation of MBP and highly significant upregulation of CNPase at 72 h post administration (Govender *et al*., 2024). At 72 h post administration, these effects are likely due to noribogaine. Early treatment effects of ibogaine are due to its affinity for sigma receptors that results in neuronal repair and remyelination (Terada *et al*., 2018; Song *et al*., 2023; Vázquez-Rosa *et al*., 2018; Yi *et al*., 2017), nicotinic alpha3beta4 receptors and NMDA that reduces neurotransmission and excitotoxicity (Wang *et al*., 2022; Kanasuwan *et al*., 2023) and the serotonin 2A receptor that has immunomodulatory effects (Yu *et al*., 2008). Its lasting effects are due to the affinities of noribogaine which remains in blood plasma for 7 days. Noribogaine has stronger affinities for the kappa opioid receptor and the serotonin transporter (Maillet *et al*., 2015; Ona *et al*.,^2023^; Mash, 2023b) which would facilitate myelin repair as well as improve mood and pain (Bulling *et al*., 2012; Du *et al*., 2016; Mei *et al*., 2016). Although the main mechanism is via neurotransmitter receptor modulation (particularly the kappa opioid, sigma 2 and NMDA receptors), secondary contributions from neurotrophic factors – such as potential modulation of myelin-associated proteins (MAG, Nogo-A) – might also support restoration of neuronal connectivity. Further research into these molecular pathways is warranted to confirm these assumptions and elucidate additional targets.

## Summary and future directions

Neurodegeneration due to toxic insult, autoimmune dysfunction or trauma requires potent pharmacological intervention to facilitate neuronal repair. White matter repair, in particular, requires potent lipophilic compounds as it requires lipid recycling and is metabolically intensive. There is a common shared white matter characteristic that is central to numerous CNS diseases that include OUD, MS, and TBI. Case reports and open-label studies of single dose ibogaine administration have documented symptom improvements, yet causality cannot be confirmed without randomised controlled trials. A possible mechanism of action may be facilitation of neuronal repair that includes remyelination which has been supported by early preclinical research. Behavioural neuroscience and preclinical studies describe additional characteristics of ibogaine that include affinity for neurotransmitter systems that target the opioidergic, serotonergic, glutamatergic and sigma receptor systems, and upregulate pro-metabolic signalling pathways. These properties of ibogaine may increase value of the drug as a treatment by supporting remyelination, restoring metabolic homeostasis and decreasing neural excitotoxicity.

In addition to randomised controlled trials to assess the safety and efficacy of ibogaine in neuropsychiatric disorders, a range of additional clinical and preclinical research would be useful in consolidating and extending the ideas raised here. Clinically, controlled and adequately powered diffusion weighted imaging studies in OUD, MS and TBI would be useful to assess if there is indeed white matter repair, increased white matter volume or white matter lesion reduction. Imaging biomarkers could include measures of white matter integrity such as fractional anisotropy, mean diffusivity and radial diffusivity or markers for myelin content such as myelin water fraction at two weeks and one month after treatment. These studies could include secondary measures of brain metabolism (using PET), cerebral blood flow (using Single-Photon Emission Computed Tomography), or Arterial Spin Labeling MRI to identify if there is a relationship between white matter repair and improved perfusion or energy metabolism.

Future studies should also utilise imaging biomarkers to investigate dose, exposure, target engagement and clinical response. There is currently a paucity of trials that have established dose range for ibogaine treatment. Mash *et al*. (2018) showed reduced symptoms of opioid and cocaine use disorders with one bolus of 8–12 mg/kg ibogaine HCl. Cherian *et al*. (2024a) showed improved function and reduced reported symptoms of PTSD, depression and anxiety following TBI with a bolus dose of 12.1 ± 1.2 mg/kg ibogaine HCl. Chen *et al*. (2025) administered 1,200 mg of ibogaine HCl to patient A with RRMS. Target dose for ibogaine that produces CNS on-target engagement and plasma and brain concentrations that modulate the desired neurotransmitter systems, is critical to establish therapeutic efficacy and avoidance of off-target toxicity. Dose-ranging and pharmacodynamic studies are therefore urgently needed to identify dosing strategies that maximise therapeutic gain while minimising risk, especially for novel indications beyond addiction.

While increased myelin protein expression after ibogaine treatment demonstrates a likely effect on oligodendrocytes, most data rely on whole-region assays – direct in vivo cell-specific effects require further study in pre-clinical demyelination models. These animal experiments could include various time points, assess axonal myelination and metabolomics markers (e.g. nicotinamide adenine dinucleotide and lipid peroxidation) in order to isolate cell-specific responses and correlate molecular, protein, and imaging outcomes with pharmacokinetic profiles. It would be valuable to compare the neuronal repair properties of ibogaine with its analogues. Many of the analogues lack the lipophilic properties of ibogaine which reduces cardiac risk but may not possess similar promyelination properties.
